# High-fat diets induce inflammatory IMD/NFκB signaling via gut microbiota remodeling in *Drosophila*

**DOI:** 10.3389/fcimb.2024.1347716

**Published:** 2024-04-23

**Authors:** Jun Wang, Jiaojiao Gu, Jianhan Yi, Jie Li, Wen Li, Zongzhao Zhai

**Affiliations:** Hunan Provincial Key Laboratory of Animal Intestinal Function and Regulation, College of Life Sciences, Hunan Normal University, Changsha, China

**Keywords:** high-fat diets, gut microbiota, *Acetobacter malorum*, IMD/NFκB, peptidoglycan

## Abstract

High-fat diets (HFDs), a prevailing daily dietary style worldwide, induce chronic low-grade inflammation in the central nervous system and peripheral tissues, promoting a variety of diseases including pathologies associated with neuroinflammation. However, the mechanisms linking HFDs to inflammation are not entirely clear. Here, using a *Drosophila* HFD model, we explored the mechanism of HFD-induced inflammation in remote tissues. We found that HFDs activated the IMD/NFκB immune pathway in the head through remodeling of the commensal gut bacteria. Removal of gut microbiota abolished such HFD-induced remote inflammatory response. Further experiments revealed that HFDs significantly increased the abundance of *Acetobacter malorum* in the gut, and the re-association of this bacterium was sufficient to elicit inflammatory response in remote tissues. Mechanistically, *Acetobacter malorum* produced a greater amount of peptidoglycan (PGN), a well-defined microbial molecular pattern that enters the circulation and remotely activates an inflammatory response. Our results thus show that HFDs trigger inflammation mediated by a bacterial molecular pattern that elicits host immune response.

## Introduction

Obesity and diabetes are growing in prevalence globally. These metabolic disorders are tightly related to diets and in turn associated with neurodegenerative diseases, particularly Alzheimer’s disease and related dementias. Recent studies indicate that high fat diets (HFD) activate early inflammation in mouse brains (Elzinga et al., 2022) and rapidly cause memory deficits (Mclean et al., 2018). In animals ingesting HFDs, an increase in inflammatory parameters and oxidative stress and a decrease in mitochondrial oxidative capacity in the brain were observed. These alterations parallel with modulation of Brain-Derived Neurotrophic Factor (BDNF), a key signaling molecule that links brain synaptic plasticity and energy metabolism (Cavaliere et al., 2019).

Inflammatory signaling is thought to play a critical role in brain functions, and there is considerable evidence linking the immune and inflammatory pathways to neurodegenerative diseases in humans (Lucin and Wyss-Coray, 2009). *Drosophila* studies also show that aberrant activation of the IMD/NFκB pathway is widely implicated in neurodegenerative diseases (Petersen et al., 2012; Cao et al., 2013; Kounatidis et al., 2017). In addition, activation of the IMD/NFκB pathway has been observed in fly head in numerous conditions, including ageing (Kounatidis et al., 2017) and ingestion of high-fat diets (Hemphill et al., 2018). The IMD/NFκB pathway in *Drosophila* is triggered by the recognition of diaminopimelic acid (DAP)-type peptidoglycan (PGN) from the cell wall of Gram-negative bacteria and *Bacillus* species by surface-bound pattern-recognition receptor PGRP-LC and cytosolic receptor PGRP-LE. Binding of PGN to the receptors initiates a signaling cascade, involving Imd, a death domain protein homologous to mammalian RIP (receptor interacting protein), the caspase 8-like protease Dredd, dTAK1 (TGF-β activated kinase 1), and the IKK complex (including Kenny (key)). This eventually leads to the activation of the NFκB factor Relish (Rel) that activates transcription of genes including those coding for secreted immune effectors and negative regulators of the pathway including the amidase PGRP-LB and PGRP-SCs that enzymatically degrade PGN thus restricting prolonged inflammation (reviewed in Zhai et al., 2018b). Antimicrobial peptides (AMPs) including *Diptericin B* (*DptB*) (Lee et al., 2001) function as the immune effectors of insects that are best known in host defense and regulation of the commensal microbiome (Hanson and Lemaitre, 2020). Recent pioneer work comprehensively dissecting the *in vivo* function of AMPs has revealed both synergy and remarkable specificity of individual AMPs in host defense (Hanson et al., 2019), and has shed light on AMP evolution driven by ecology-relevant bacteria (Hanson et al., 2023). In addition, AMPs were also linked to the maintenance of long-term memory in *Drosophila* (Barajas-Azpeleta et al., 2018). Overexpression of individual AMP genes either in neurons or glia was sufficient to trigger neurodegeneration, pointing to a cytotoxic effect of high levels of AMPs and inflammation to the fly brain (Cao et al., 2013).

Mechanisms linking HFDs and immune activation remain elusive. Commensal gut microbiota exerts profound effects on both the intestinal and systemic immune homeostasis. A disruption in the microbial composition of the gut has been associated with many neurological disorders with inflammatory components in mammals (Janakiraman and Krishnamoorthy, 2018). Such neuroinflammation is a common feature of virtually every central nervous system (CNS) diseases, and is being increasingly recognized as a mediator of cognitive decline and neurodegenerative diseases (Kumar, 2018). As gut microbiota is deeply impacted by environmental factors such as diets (Rothschild et al., 2018), it is interesting to define how diets and microbiota interact to shape neuroinflammation. Microbial metabolites modulate CNS inflammation (Rothhammer et al., 2016). For instance, bacterial PGN derived from the commensal gut microbiota can translocate into the brain via circulation where it is sensed by specific pattern-recognition receptors of the innate immune system. One interesting study showed that the absence of a PGN-recognition receptor, *Pglyrp2*, led to alterations in the expression of the autism risk gene and sex-dependent changes in social behaviour, reminiscent of mice with manipulated microbiota (Arentsen et al., 2017), thus suggesting communication between the gut microbiota and the developing brain.

In this study, we aim to establish a causal link of HFDs to remote inflammation using the genetically tractable animal model *Drosophila melanogaster*. This led to the identification of a critical role of the gut microbiota remodeling in shaping remote inflammatory response.

## Results

### HFD triggers remote immune response in the fly head

**Figure 1:**
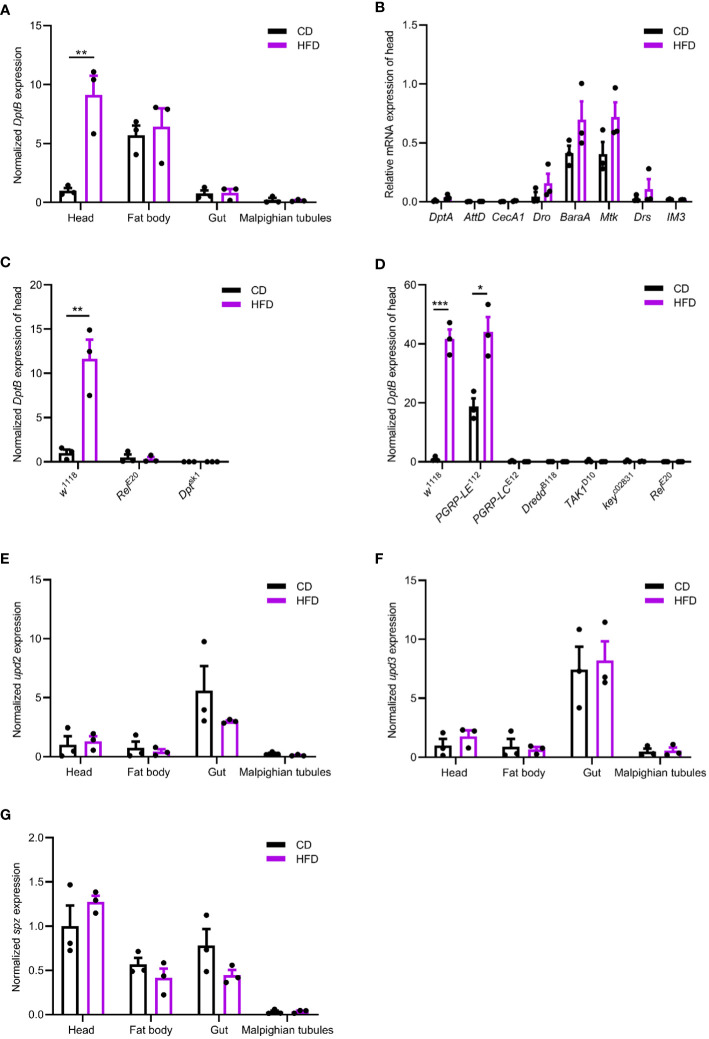
HFD remotely activates the IMD/NFκB immune pathway. **(A, E–G)** Normalized *DptB* **(A)**, *upd2* **(E)**, *upd3* **(F)** and *spz* **(G)** mRNA levels in the head, the fat body, the gut and the Malpighian tubules of flies raised on CD and HFD for 5 days. **(B)** Normalized *DptA*, *AttD*, *CecA1*, *Dro*, *BaraA*, *Mtk*, *Drs* and *IM3* mRNA levels in the head of flies raised on CD and HFD. **(C, D)** Normalized *DptB* mRNA levels in the head of wild type control (*w^1118^*), *Relish* mutant (*Rel^E20^*), *Dpt* mutant (*Dpt^sk1^*), *PGRP-LE* mutant (*PGRP-LE^112^*), *PGRP-LC* mutant (*PGRP-LC^E12^*), *Dredd* mutant (*Dredd^B118^*), *TAK1* mutant (*TAK1^D10^*) and *Kenny* mutant (*key^c0283^* ^1^) flies raised on CD and HFD. Data are presented as mean ± SEM from three independent experiments. Each dot represents an independent replicate. Statistical significance is presented as *p < 0.05, **p < 0.01, ***p < 0.001.

By adding 30% coconut oil into our conventional fly diet (CD), a high-fat diet was used to feed flies for 5 days before the expression of IMD/NFκB activity readout, *DptB*, was measured. *DptB* expression in the midgut did not show any difference between flies raised on CD and HFD (**Figure 1A**), suggesting that the local gut immunity was not altered by a HFD. In addition, the abdominal fat body and the Malpighian tubules did not show upregulation of *DptB* upon HFD either. Interestingly, we found that the fly head, which includes the brain, eyes, head cuticles and head fat bodies that interiorly attach to the cuticle, consistently upregulated *DptB* expression (**Figure 1A**). *DptB* is expressed in the head fat body cells as previously shown using a reporter gene (Barajas-Azpeleta et al., 2018), suggesting remote activation of IMD signaling in the fat tissue of the head by HFD. mRNA levels of several additional immune effectors including *DptA, AttD, CecA1, Dro, BaraA, Mtk, Drs and IM3* (*BomS3*) were also measured from the head of flies raided on CD and HFD, and none of them differed significantly between the two diet conditions (**Figure 1B**). Thus, HFD appears to specifically induce *DptB* but not other effectors only in the head fat body.

Using a *Relish* null mutant, we found HFD-induced activation of *DptB* was completely Relish-dependent (**Figure 1C**). To further study if HFD-induced activation of *DptB* was mediated by the canonical IMD/NFκB pathway, a number of other flies mutant for key components of the IMD pathway were analyzed. In mutants with blocked IMD signal transduction (*TAK1*, *Key* and *Dredd* mutants), HFD-induced *DptB* upregulation was totally abolished as that in *Relish* mutant flies (**Figure 1D**). Interestingly, the induction of *DptB* by HFD required the pattern recognition receptor PGRP-LC that locates to the plasma membrane but not the cytosolic PGN receptor PGRP-LE. Of note, the higher level of *DptB* expression in the head of *PGRP-LE* mutant flies raised on CD is consistent with a recent observation that gut-specific depletion of *PGRP-LE* caused systemic immune activation during gut infection due to the inability to control gut bacteria (Joshi et al., 2023). Taken together, HFD upregulated fat body immunity dependent on the canonical IMD pathway.

To rule out the possibility that HFD causes damages to the gut epithelium and consequently makes a leaky gut, we first measured the expression of three proposed fly cytokines (Unpaired 2 (Upd2), Upd3 and Spaetzle (Spz)) (Woodcock et al., 2015; Yu et al., 2022) in the midgut, the head, the abdominal fat body and the Malpighian tubules of flies raised under CD and HFD, but again failed to detect any difference (**Figures 1E–G**). Furthermore, Smurf assay measuring gut permeability (Rera et al., 2012) also argued against a general defect in the integrity of the intestinal epithelium as we did not detect any positive individuals with pathological gut permeability in both CD and HFD conditions (data not shown). These results reinforced that instead of causing an immune activation or epithelial damages locally in the gut, HFD led to inflammation remotely.

### HFD promotes remote inflammation via microbiota remodeling

**Figure 2:**
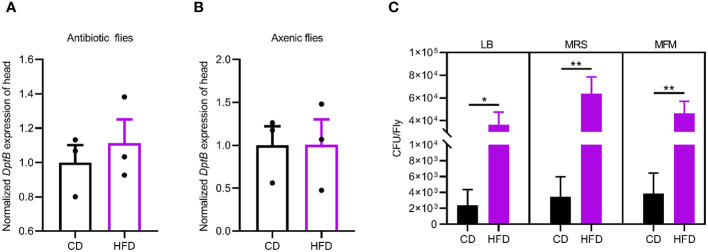
HFD-induced gut microbiome remodeling is required for remote immune activation. **(A, B)** *DptB* mRNA levels in flies with gut bacteria removed either via an antibiotic cocktail in the adult **(A)** or using bleaching method to make axenic flies **(B)**, raised on CD and HFD. Note that microbe-less flies did not show immune activation in HFD condition. **(C)** The bacterial load (CFUs) in flies raised on CD and HFD. Culturing media used were Luria-Bertani medium (LB), Man-Rogosa-Sharpe medium (MRS) and Mannitol Ferment Medium (MFM). Data are presented as mean ± SEM from three independent experiments in A and B and from six independent experiments in C. Statistical significance is presented as *p < 0.05, **p < 0.01.

The IMD pathway is activated by sensing bacterial PGN. We therefore hypothesized that changes in the gut microbiota may underline the inflammatory response to the HFD in fly head. To test this idea, we removed the gut bacteria either by feeding flies a cocktail of antibiotics in the adult stage or by generating axenic flies that are not confronted with any microbes from birth onwards (Guo et al., 2014). We first confirmed both methods efficiently eliminated bacteria from flies (data not shown). Then, such microbe-less flies were raised either on CD or on HFD conditions and measured for *DptB* expression in the head. We found that *DptB* induction by the HFD was totally repressed using both methods, pointing to a role of gut microflora in causing inflammation in remote tissues (**Figures 2A, B**).

**Figure 3:**
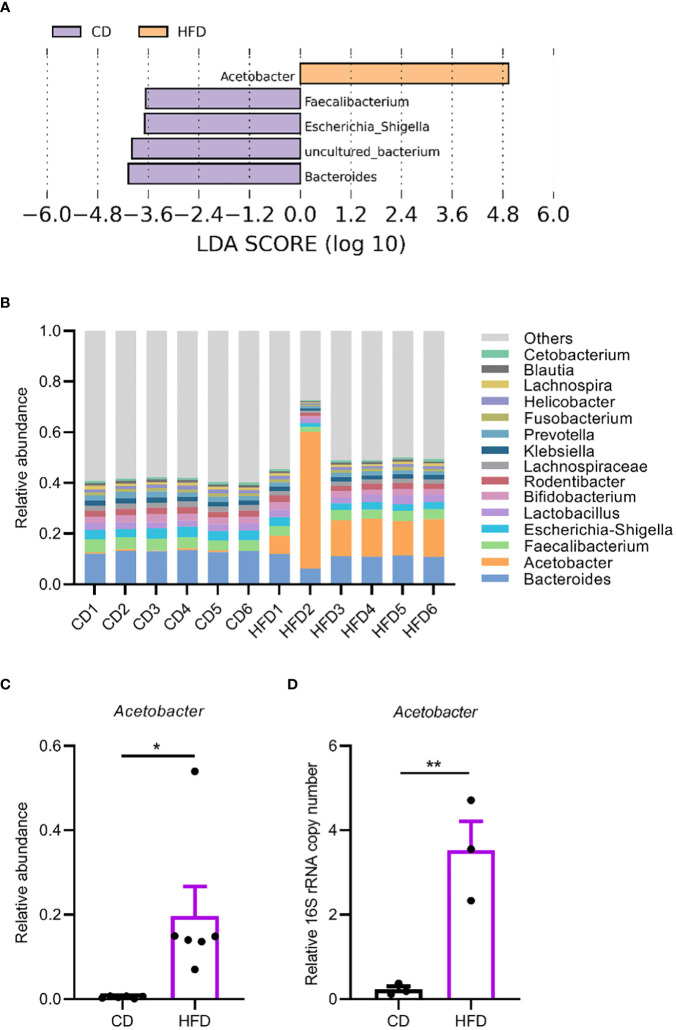
HFD upregulates *Acetobacter* in the gut microbiome. **(A, B)** Linear discriminant analysis Effect Size (LEfSe) to determine differentially enriched bacteria in flies fed CD or HFD. **(A)** and relative abundance of bacteria in the genus level in flies fed a CD or a HFD measured with 16S amplicon sequencing **(B)**. Six biological replicates were performed for both CD and HFD conditions. **(C)** The abundance of *Acetobacter* from the 16S amplicon sequencing. **(D)** The relative bacterial load analyzed by qPCR using *Acetobacter* species-specific primers. Data are presented as mean ± SEM from three independent experiments in **(D)**. Each dot represents an independent replicate. Statistical significance is presented as *p < 0.05, **p < 0.01.

Then we sought to detect changes in the gut microbiota due to HFD. We quantified overall microbial load by measuring colony-forming units (CFUs) in flies raised on CD and HFD, using selective plates to identify Lactobacillae (MRS), Acetobacteriaceae (MFM), and bacteria grown on nutrient-rich medium (LB). The bacterial load increased in flies raised on HFD compared to CD in all the three culturing media (**Figures 2C**). This implies a general increase in bacterial load. To further dissect the microbiota structure, 16S metagenomic analyses were performed using six biological replicates for flies raised on each culturing conditions concerning the potential variability of the microbiome. This analysis uncovered that our HFD significantly altered the composition of the microbial community in comparison with that of flies raised on a CD. Linear discriminant analysis Effect Size (LEfSe) method revealed that *Acetobacter* was the most enriched genus upon HFD feeding. On the other hand, flies fed a CD exhibited enrichment of species belonging to *Faecalibacterium*, *Escherichia*, *Bacteroides* as well as some *uncultured bacteria*. To our surprise, while most of the bacterial genera showed similar relative abundance in CD and HFD conditions, *Acetobacter* species consistently increased their composition in the gut microflora of flies fed with the HFD (**Figures 3A, B**). This made *Acetobacter* the top bacterium in HFD condition. We further validated the increase of *Acetobacter* species using quantitative PCR measuring 16S rRNA regions specific for *Acetobacter* and identified a 15-fold increase in the amount of *Acetobacter* (**Figures 3C, D**). Thus, *Acetobacter* strongly increased their abundance in the intestine of flies raised on our HFD.

Laboratory-reared *Drosophila melanogaster* harbors a simple gut microbiome with only several dominating bacterial species that belong to the *Lactobacillus* and *Acetobacter* genera (Broderick et al., 2014). However, we noticed that even in our CD condition, the abundance of *Lactobacillus* and *Acetobacter* species in our laboratory were generally very low, potentially suggesting a strong impact of diets and environmental factors on the structure of gut microbiome (Martino et al., 2017).

### *Acetobacter malorum* recapitulates HFD-induced inflammation by increasing circulating PGN

**Figure 4:**
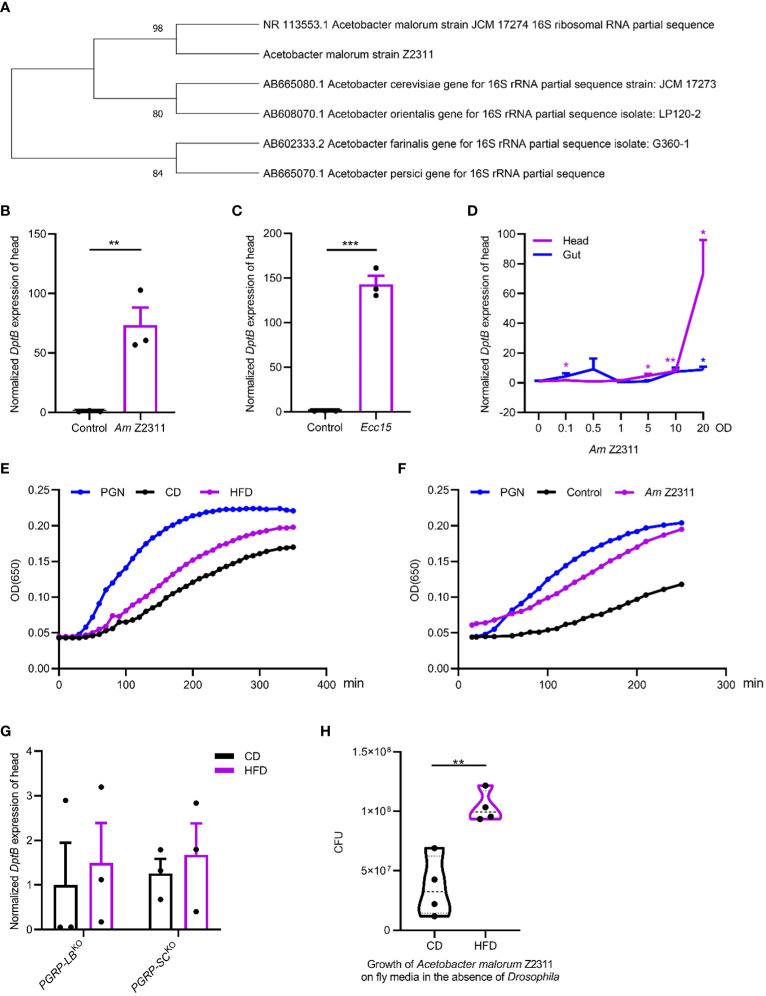
*Acetobacter malorum* is sufficient to induce remote inflammation in head via increasing circulating PGN. **(A)** The phylogeny based on 16S rRNA sequence indicating the relationship of *Acetobacter malorum* Z2311 (*Am* Z2311) isolated in this study with several most closely related *Acetobacter* species. **(B, C)** *DptB* mRNA levels in the head of flies fed with *Am* Z2311 **(B)** or *Ecc15* **(C)** on a conventional diet. **(D)** Normalized *DptB* mRNA levels in the head and the gut of flies ingesting *Am* Z2311 of increasing concentrations. **(E, F)** Hemolymph PGN quantification from 100 female adults in each condition using enzymatic kinetics with 100ng/mL PGN as positive control. Note that flies were either raised on CD and HFD conditions **(E)**, or raised on a CD condition but re-associated with the *Am* Z2311 strain **(F)**. Each figure is the presentative result of three independent measurements. **(G)** *DptB* mRNA levels in the head of flies lacking amidase PGRPs*, PGRP-LB^KO^* and *PGRP-SC^KO^* raised on CD and HFD conditions. Note that HFD did not further increase the head inflammation in both *PGRP-LB^KO^* and *PGRP-SC^KO^* flies. **(H)** The bacterial load (CFUs) of *Am* Z2311 after equal amount of bacteria being cultured on CD and HFD fly media for 2 days without the presence of *Drosophila*. Data are presented as mean ± SEM from three independent experiments. Each dot represents an independent replicate. Statistical significance is presented as *p < 0.05, **p < 0.01, ***p < 0.001.

We next sought to identify the *Acetobacter* species nourished by the HFD and see if it underlies HFD-induced inflammation in remote tissues. By plating on mannitol agar serial dilutions of homogenized gut of flies raised on HFD, uniform colonies with similar morphological features were seen. Ten representative isolates were cultured and characterized by PCR amplification followed by sequencing of the 16S rRNA gene. We found that they all represented the same bacterial species, with the closest relationship to a previously isolated *Acetobacter malorum* strain JCM 17274 using BLAST search (**Figure 4A**). The JCM 17274 strain can only be traced back to the following study (Cleenwerck et al., 2002) without detailed information whether it belongs to a gut commensal of flies. We named our strain as *Acetobacter malorum* Z2311. To test if the *Acetobacter malorum* Z2311 strain mimicked HFD-induced effects, we mono-associated axenic flies with our Z2311 strain and found this strain produced a strong upregulation of *DptB* in the head, to a comparable level of *Ecc15* oral infection (**Figures 4B, C**). *Ecc15* belongs to the phytopathogenic bacterial genus *Erwinia* (now *Pectobacterium*) and is a well characterized strain used to induce fly immune response (Buchon et al., 2009b; Zhai et al., 2018a). In addition to local gut immune response, oral *Ecc15* infection also activates a systemic immune response that is dependent on the IMD pathway (Basset et al., 2000). To see if oral ingestion of the Z2311 strain resulted in an infection-like condition, flies were fed with bacteria of different concentrations ranging from OD_600_ 0 to 20, and checked for *DptB* expression in both the gut and the head. While *Acetobacter malorum* Z2311 had only very mild induction of *DptB* in the gut across all the concentrations tested, it induced *DptB* expression for around 70 folds in the head at OD_600_ 20 (**Figure 4D**). This implies that the *Acetobacter malorum* Z2311 bacteria trigger only very limited local gut immune response thus remain largely unnoticed by the gut epithelium, but instead are competent to induced remote *DptB* expression in the head.

Gut-bacteria-derived PGN can translocate into the hemolymph that bathes most adult organs and tissues and activate the IMD/NF-κB pathway remotely (Charroux et al., 2018). We wondered if HFD-induced elevation of *Acetobacter malorum* Z2311 activated immune response in remote tissues by increasing PGN release into the circulation. Silkworm Larvae Plasma (SLP) assay was used to quantify circulating bacterial PGN in the hemolymph (Troha et al., 2019). We found that the amount of circulating bacterial PGN was significantly higher in flies raised on HFD than on CD (**Figure 4E**). Furthermore, mono-association of flies with *Acetobacter malorum* Z2311 significantly increased circulating bacterial PGN (**Figure 4F**). Further supporting the role of PGN in promoting remote immune activation, flies carrying deletions each removing key amidase PGRPs (PGRP-LB and PGRP-SCs) showed higher *DptB* expression already under CD, but did not further increase their immune activation under HFD (**Figure 4G**). This is consistent with the notion that the inability to remove PGN locally from the gut and systemically form the circulation renders flies to uncontrolled immune activation. Finally, to better dissect the host-microbe interactions in a nutritional environment, we have measured the growth of *Acetobacter malorum* Z2311 by directly culturing them in both control and HFD fly media in the absence of *Drosophila*. Interestingly, the Z2311 strain grew much faster on HFD than on control diet (**Figure 4H**), supporting that Z2311 itself is better adapted to the high-fat environment. It remains interesting to test in future whether this *Acetobacter malorum* strain also impacted the host adaptation to the HFD condition. In sum, our study supports that HFD-induced changes in the gut microbiome increased the systemic release of PGN that remotely activates immune response.

## Discussion

Reminiscent of human studies, HFDs have been reported to cause a variety of pathologies in *Drosophila*. HFD-fed flies exhibited increased levels of triglyceride and alterations in insulin/glucose homeostasis (Heinrichsen et al., 2014). A HFD also caused cardiac lipid accumulation, reduced cardiac contractility and severe structural pathologies, reminiscent of diabetic cardiomyopathies (Birse et al., 2010). In addition, flies fed a lipid-rich diet presented a systemic activation of JAK/STAT signaling, reduced insulin sensitivity, hyperglycemia, and a shorter lifespan, features that were all dependent on a macrophage-Unpaired 3 axis (Woodcock et al., 2015). Unpaired 3 has a counterpart in mammals called Interleukin-6, which has previously been associated with diseases induced by HFDs in mice (Han et al., 2013). With this work, we added a mechanistic link between HFD-induced inflammation and gut microbiota remodeling using *Drosophila* model. Our study unexpectedly identified a single bacterium that became exceptionally enriched in the gut microbiome of animal ingesting a HFD, and showed this bacterium has a remarkable ability to remotely activate immune response through releasing its cell wall components, the PGN, into the circulation. Remarkably, the specific induction of *DptB* but not other AMPs by HFDs, a condition that we showed in this work to greatly enrich the *Acetobacter* bacterium Z2311, is in concert with a recent work showing that *DptB* is specifically required for defense against *Acetobacter* bacteria during AMP evolution driven by ecology-relevant microbes (Hanson et al., 2023).

Gut microbiota is not essential for development and survival of *Drosophila*, but resident bacteria profoundly impact fly physiology and behaviors (Schretter et al., 2018; Zhai et al., 2018b; Jia et al., 2021). Gut dysbiosis, which is characterized by a loss of normal bacterial community structure and an increase in bacterial load, has been considered a major risk factor contributing to intestinal pathology and organismal ageing (Buchon et al., 2009a; Guo et al., 2014; Clark et al., 2015; Li et al., 2016; Zhou and Boutros, 2020; Chen et al., 2022). Enteric infection was reported to remotely exacerbate the progression of Alzheimer’s disease in a *Drosophila* model (Wu et al., 2017). Thus, while normal microbiota provides the host with essential immune and metabolic benefits, its deregulation contributes to the initiation/progression of diseased states. Lactic and acetic acid bacteria are key gut microbiome members in flies (Broderick, 2016). *Acetobacter* commensals significantly impact *Drosophila* development (Shin et al., 2011; Consuegra et al., 2020a, b), food choice and reproduction (Elgart et al., 2016; Leitao-Goncalves et al., 2017), metabolism and behaviors (Newell and Douglas, 2014; Fischer et al., 2017), and lifespan (Obata et al., 2018; Onuma et al., 2023). Of note, a recent work interestingly uncovered the significance of commensal bacterial PGN specificity in determining the gut bacterial impact on the immune activation (Onuma et al., 2023). *Acetobacter* and its PGN activated *DptA* expression in the gut while the *Lactobacillus* strain and its PGN had much weaker potency to do so. Together with our results that HFDs led to a specific overrepresentation of *Acetobacter* in the microbiome and caused an inflammatory response in remote tissues, it appears that PGN from *Acetobacter* acts as a strong elicitor of systemic immune response.

However, we still do not completely understand the underlying mechanisms of HFD-induced remote immune activation that our study found unique at least in the following three ways. First, HFD-induced *DptB* activation was restricted to the fat body in the head but did not occur in the other systemic tissues examined. This seems inconsistent with the role of circulatory PGN as a bacteria-derived inflammatory molecular pattern, but varied sensitivity of respective tissues to PGN may be at work. Differences in gene expression and function between the head fat body and abdominal fat body were reported previously (Benes et al., 1990; Sun et al., 2017; Barajas-Azpeleta et al., 2018), likely reflecting their developmental origin. The anterior portion of the larval fat body becomes encapsulated within the head following head eversion, and possibly undergoes limited subsequent histolysis during insect metamorphosis, contrasted to the fat body cells located in the abdominal segments where the remaining larval fat body cells are fully replaced during young adult stage (Jowett and Postlethwait, 1981; Benes et al., 1990). Therefore, we speculate that the head fat body retains features of larval fat cells, but this requires verification experimentally. Second, *DptB* but not other genes encoding AMP examined in this study was specifically upregulated by HFD. This may suggest different levels of IMD activation required for respective AMP to be induced, while we cannot exclude the possibility that *DptB* is induced by specific environmental conditions. Indeed, various IMD outputs do not always show uniform alterations in response to genetic and microbial factors (Bonnay et al., 2014; Onuma et al., 2023). Third, it is striking that the *Acetobacter* bacterium isolated in this study barely altered the local *DptB* expression profile of the gut while it acted as a competent inducer of *DptB* remotely in the head. It will be interesting to check in future if this *Acetobacter* species sheds PGN mainly in the polymeric form that is not sensed by PGRP-LE-dependent epithelial immunity but activates PGRP-LC-dependent systemic immunity in the head fat body (**Figure 1D**) (Bosco-Drayon et al., 2012; Neyen et al., 2012).

It is now well accepted that HFDs induce a systemic chronic low-grade inflammation in both the CNS and peripheral tissues in mammals (Duan et al., 2018). High fat consumption also causes overproduction of circulating free fatty acids. It was hypothesized that alterations in the gut microbiota triggered by HFDs as well as the direct effects of free fatty acids on intestinal cells may be the initial step in causing systemic inflammation. Indeed, germ-free mice exhibited neither obesity nor overproduction of inflammatory cytokines in the intestine even on HFDs (Backhed et al., 2004; Turnbaugh et al., 2009). On the other hand, a higher bacterial diversity in the gut microbiome negatively correlated with the possibility to develop adiposity and inflammation in a human cohort study (Le Chatelier et al., 2013). Increased amounts of free fatty acids from HFDs may directly act on intestinal epithelial cells to modify intestinal permeability, enhancing the translocation of microbial metabolites such as LPS and PGN across the gut epithelium and to the circulation. This in turn activates the immune cells through pattern recognition receptors that further promote the production of proinflammatory cytokines (Ding et al., 2010; Kim et al., 2012; Wu et al., 2017). Thus, the gut microbiota, free fatty acids, the immune cells, and circulating cytokines likely act collectively downstream of HFDs to cause a systemic low-grade inflammation in remote tissues. Indeed, circulating cytokines and microbial molecular patterns have been reported to reach the hypothalamus and initiate inflammation through processes such as microglial proliferation (Rothhammer et al., 2016; Tan and Norhaizan, 2019; Hersey et al., 2021; Elzinga et al., 2022). Here, a similar mechanism is likely operating in flies ingesting a HFD, in which HFD changes the gut microbiota likely due to a metabolic adaption by the host and the gut microbes to the potential higher amount of free fatty acids or other lipid metabolites, and further indirectly alters NFκB activity remotely in the head.

We now know that the host-microbe interactions are largely shaped by the nutritional environment (Martino et al., 2017). Our data support that *Acetobacter malorum* can better utilize the rich nutrients contained by HFDs and therefore thrives in such nutrient-rich environment, with a similar mechanism as reported before (Martino et al., 2018). However, it is currently unclear if the increase in *Acetobacter malorum* benefits the host by promoting metabolic adaption to HFDs. As nutritional environment dominates over host genetics in shaping human gut microbiota (Rothschild et al., 2018), in future, it will be interesting to determine the specific changes of gut microbiota caused by HFDs that are related to the increased neuroinflammation in humans.

## Materials and methods

### *Drosophila* husbandry

Fruit flies were cultured in 21°C and under 60% humidity with a 12:12 light dark cycle. Conventional fly diet (CD) contains per liter 32.3g yeast, 69.2g corn flour, 9.2g soybean meal, 61.5mL syrup, 1.7g Nipagin methyl ester and 7.7g Agar. The high-fat diet (HFD) was made by adding into CD 30% coconut oil (*w/v*). Unless otherwise noted, flies were cultured on CD or HFD for 5 days before they were analyzed. Fly strains used in study are isogenic *w^1118^* as wild type, *Rel^E20^*, *PGRP-LB^KO^*, *PGRP-SC^KO^*, *PGRP-LE^112^*, *PGRP-LC^E12^*, *Dredd^B118^*, *TAK1^D10^*, *key^c02831^* and *Dpt^sk1^*.

### Axenic flies

Flies were allowed to lay eggs on apple juice agar plates (containing 2% agar and 50% apple juice) for 4 hours at 25˚C. The eggs were then collected with deionized water using a brush and net baskets. We washed the eggs three times with 70% ethanol, and disinfected them with bleach diluted with deionized water. The eggs were then washed thoroughly with deionized water before being transferred to sterilized food supplemented with an antibiotic cocktail (500μg/mL Ampicillin; 50μg/mL Tetracycline; 200μg/mL Rifamycin) used previously (Jia et al., 2021). To make microbe-less flies starting only from the adulthood, flies were conventionally raised to 3-5 days post eclosion, and shifted to food supplemented with the antibiotic cocktail mentioned above. The axenic state of flies was confirmed by culturing method.

### Smurf assay

Flies were cultured on CD with blue dye (Sigma, UAS, Cat. #861146) for 1 day after they were treated on CD or HFD for 5 days. Smurf flies that have a leaky gut were recorded as those exhibiting a visible blue color throughout their hemocoel within the body cavity (Rera et al., 2012).

### Colony-forming units assay

CO2 anesthetized flies were surface cleaned with 70% ethanol and homogenized in group of 15 flies with the Bertin Precellys Evolution Homogenizer. Then, their homogenates were serially diluted and plated on plates made with either Luria-Bertani medium (LB), Man-Rogosa-Sharpe medium (MRS) or Mannitol Ferment Medium (MFM). After the plates were cultured in 29°C for 48 hours, colonies were manually counted and calculated as CFU/fly.

To check bacterial growth rate in the CD and HFD fly media in the absence of *Drosophila*, 40μL (OD_600 _0.001) *Acetobacter malorum* Z2311 was added to sterilized CD and HFD food tubes and cultured at 21°C for 2 days. The tubes were washed with 1mL sterile water that was serially diluted and plated on LB plates. The plates were cultured at 29°C for 48 hours, and then colonies were manually counted and calculated as CFU/tube.

### Isolation of *Acetobacter malorum* strain Z2311

Homogenate of fruit flies fed with HFD was cultured on selective MFM medium for *Acetobacter*. Multiple single colonies were picked, cultured and sequenced using universal primers (27F: 5’-AGAGTTTGATCCTGGCTCAG-3’ and 1429R: 5’-GGTTACCTTGTTACGACTT-3’) for the bacterial 16S rRNA to confirm their identity. The sequences of our isolated *Acetobacter malorum* strain (accession number: PRJNA1063874) were Blast searched, and the phylogenetic tree was drawn together with the most closely related *Acetobacter* species using MEGA11. For re-association experiment, *Acetobacter malorum* Z2311 was orally fed to flies for 12 hours at concentrations of OD_600_ 0.1, OD_600_ 0.5, OD_600_ 1, OD_600_ 5, OD_600_ 10 and OD_600_ 20. In another experiment, *Acetobacter malorum* Z2311 and *Ecc15* were orally fed to flies for 12 hours at the concentration of OD_600_ 20. The feeding of bacteria was done essentially via soaking into a filter paper fully covering fly food as previously described (Zhai et al., 2018a).

### Hemolymph PGN detection

Hemolymph collection and PGN quantification were done essentially according to a recent paper (Chen et al., 2022). Briefly, to collect hemolymph, 100 decapitated female adults were centrifuged at 1500*g* for 15min at 4°C, followed by a 5 min heating at 70°C. The supernatant was collected and further centrifugated at 12000*g* for 10 min at 4°C. The extracted hemolymph was 1:10 diluted before PGN quantification. SLP-HS Single Reagent Set II (Fujifilm Wako Pure Chemical Corporation, Japan, Cat. #296-81001) was used to detect PGN in the hemolymph according to the manufacturer instructions.

### 16S rRNA amplicon sequencing

Whole flies were used for 16S rRNA sequencing. Flies were surface cleaned before being processed further. Bacterial DNA was co-extracted with fly genomic DNA and amplified with primers (343F: 5’-TACGGRAGGCAGCAG-3’; 798R: 5’-AGGGTATCTAATCCT-3’) specific for bacterial 16S rRNA gene (V3/V4 region). Sequencing library preparation and MiSeq (Illumina) high-throughput sequencing were done in oebiotech (Shanghai, China). Sequencing data were processed using standard procedure and raw sequencing data are available under BioProject ID PRJNA1047347.

### Quantitative PCR

Total RNA was extracted from the head, the fat body, the gut and the Malpighian tubules of 15-20 flies using RNAiso Plus (TaKaRa, Dalian, China, Cat. #9109). cDNA was synthesized using the PrimeScript RT reagent Kit (TaKaRa, Dalian, China, Cat. #RR037A). 0.2-0.5μg total RNA was used for reverse transcription with oligo (dT), and the 1^st^ strand cDNA was diluted 10-20 times with water and further used in real time PCR. Real time PCR was performed in at lease duplicate for each sample using LightCycler 480 SYBR Green (Roche, Switzerland, Cat. #04887352001) on a q225 qPCR System from Quantagene (Kubo Technology, Beijing, China). Bacterial DNA contained in the fly gut was extracted using a Bacteria DNA Kit (TIANGEN, China, Cat. #DP302). The expression value was calculated by ΔΔ Ct method, and the relative expression was normalized to *RpL32* or *GAPDH*. Primers used are shown in **Table 1**.

**Table 1:** Sequence of primers used for qPCR.

Genes	Sequence
*RpL32_F*	TCTGCATGAGCAGGACCTC
*RpL32_R*	ATCGGTTACGGATCGAACAA
*DptA_F*	GCGCAATCGCTTCTACTTTG
*DptA_R*	CCTGAAGATTGAGTGGGTACTG
*DptB_F*	ACTGGCATATGCTCCCAATTT
*DptB_R*	TCAGATCGAATCCTTGCTTTGG
*Drs_F*	AAGTACTTGTTCGCCCTCTTC
*Drs_R*	CACAGGGACCCTTGTATCTTC
*BaraA_F*	GGTAATGGCGGCGTCTATATT
*BaraA_R*	AGCCACCGTTACCGAAATC
*CecA1_F*	CTCAGACCTCACTGCAATATCAA
*CecA1_R*	CCAGAATGAGAGCGACGAAA
*Mtk_F*	GCAACTTAATCTTGGAGCGATTT
*Mtk_R*	GGTCTTGGTTGGTTAGGATTGA
*AttD_F*	GTATACCTCTCCAAGTGGCAATC
*AttD_R*	TTAACTCCGGTGCCGAAATC
*IM3_F*	GGTACACTTGGCTGCTCTATG
*IM3_R*	GCTTGACTCCCGCGTATTAG
*Dro_F*	TCGAGGATCACCTGACTCAA
*Dro_R*	GATGACTTCTCCGCGGTATG
*Upd2_F*	ACCATTGCTGTTCGGATAGG
*Upd2_R*	AGCAGAAGAGCCTCAACGAG
*Upd3_F*	CCAGAACCAGGAATCCAGTG
*Upd3_R*	GCCAAGGCGAGTAAGATCAG
*Spz_F*	CTCAGACGAGCGATTCCTTT
*Spz_R*	TGGCCTGTTTGTACTCATCG
*GAPDH_F*	TAAATTCGACTCGACTCACGGT
*GAPDH_R*	CTCCACCACATACTCGGCTC
*Acetobacter_F*	TAGTGGCGGACGGGTGAGTA
*Acetobacter_R*	AATCAAACGCAGGCTCCTCC

### Statistical analysis

Data are shown as means ± SEM from at least three replicates of each experiment. Statistical significance between two groups was calculated with Unpaired Student’s *t* test to assess differences using GraphPad Prism 9. Statistical significance is presented as *p < 0.05, **p < 0.01, ***p < 0.001.

## Data availability statement

The datasets presented in this study can be found in online repositories. The names of the repository/repositories and accession number(s) can be found below: BioProject ID: PRJNA1047347.

## Ethics statement

The manuscript presents research on animals that do not require ethical approval for their study.

## Author contributions

JW: Conceptualization, Data curation, Formal analysis, Investigation, Methodology, Project administration, Software, Validation, Visualization, Writing – original draft. JG: Investigation, Methodology, Writing – original draft. JY: Investigation, Methodology, Writing – original draft. JL: Formal analysis, Investigation, Methodology, Writing – original draft. WL: Investigation, Writing – original draft. ZZ: Conceptualization, Funding acquisition, Project administration, Resources, Supervision, Writing – original draft, Writing – review & editing.
